# Natural Copper Ion Scavenger: Investigation of the Hepatoprotective Effects of Green Tea Extract in Toxic-Milk Mice with Wilson’s Disease Model

**DOI:** 10.3390/foods14040679

**Published:** 2025-02-17

**Authors:** Delai Yang, Shujuan Xuan, Wang Zhang, Huan Wu, Yuge Jiang, An Zhou

**Affiliations:** 1 School of Pharmacy, Anhui University of Chinese Medicine, Hefei 230038, China; ydl11011671@163.com (D.Y.); 13855108231@163.com (S.X.); 2020205221006@stu.ahtcm.edu.cn (W.Z.); 2 Key Laboratory of the Ministry of Education of Xinan Medicine, Hefei 230038, China; wuhuancpu@ahtcm.edu.cn; 3 Anhui Province Key Laboratory of Research and Development of Chinese Medicine, Functional Activity and Resource Utilization on Edible and Medicinal Fungi Joint Laboratory of Anhui Province, Hefei 230038, China

**Keywords:** Wilson’s disease, green tea extract, green tea polyphenols, L-theanine, decoppering, antioxidation

## Abstract

Wilson’s disease (WD) is an inherited disorder characterized by abnormal copper metabolism with complex pathological features. Currently, the mechanism of copper overload-induced hepatic injury is unclear. Green tea is a natural chelator, and its main ingredients, green tea polyphenol (GTP) and L-theanine (L-TA) are good at binding to heavy metals like iron and copper. There have been no reports on green tea extracts (GTE) for the treatment of Wilson’s disease. This study investigated the hepatoprotective effect of GTE on WD model mice. Initially, we examined the impact of green tea extract on copper metabolism, excretion, and hepatoprotective effects in WD model toxic milk mice. Then, Ultra performance liquid chromatography (UPLC-DAD) was established to analyze GTP and L-TA in green tea extract. Further screening of eight active components and copper complex active components in green tea extract was carried out by ion analyzer. Finally, we verified the pharmacodynamic effects of these active ingredients at the animal level. The results showed that GTE improves liver function and attenuates liver injury in TX mice by promoting tissue copper excretion and inhibiting oxidative stress, which provides a theoretical basis for green tea’s potential to improve the clinical symptoms of WD.

## 1. Introduction

Wilson’s disease (WD) is an autosomal recessive ailment caused by *ATP7B* gene mutations [1]. WD is characterized by poor copper metabolism; excess copper accumulation can be harmful to the liver, kidneys, and brain, endangering these patients’ lives [2,3]. Copper is an essential trace metal in the human body; it is an active constituent in many enzymes and plays a role in a variety of physiological processes, including energy metabolism and redox reactions [4,5,6]. Overaccumulation of copper in the mitochondria releases several reactive oxygen species (ROS), leading to the breakdown of mitochondrial membranes and death of hepatocytes [7]. Another study found that copper accumulation in WD patients in the early stages of the disease could induce modest liver damage, such as hepatocyte edema, local distortion and enlargement of mitochondria, and so on. When the disease shows up late, it can lead to much copper building up in the body, which can destroy hepatocytes, cause necrosis and inflammation, and lead to enlarged nuclei, protruding nucleolus, vacuolization, and other problems [8,9]. The most successful clinical treatment for WD is copper chelation therapy. This entails utilizing copper chelating compounds like D-penicillamine (PCA) and tetrathiomolybdate (TM) to help eliminate copper from the body. Unfortunately, they are frequently associated with a variety of hazardous side effects, such as immunologically caused lesions, renal damage, and poor patient compliance, all of which can contribute to therapy failure [10,11]. As a result, there is a need to develop more potent drugs with fewer side effects in order to treat WD.

Green tea has a high antioxidant content and regulates the body’s balance of oxygen-free radicals and free radical scavengers. Green tea is the most natural metal chelator, with antioxidant and metal complexing properties observed in both in vivo and in vitro experimental models [12,13]. Previous research has also shown that green tea can complex metal ions and has strong antioxidant properties [14]. This means that it can protect the liver of mice from oxidative damage caused by carbon tetrachloride, alcohol, and other hepatotoxic substances [15,16]. A prior survey indicates that workers frequently exposed to heavy metals like lead, copper, and iron are more susceptible to heavy metal poisoning. However, the overall disease incidence is lower, and the symptoms are milder for people who drink tea on a regular basis. Previous research has also demonstrated that patients with WD who drink tea have lower levels of copper in their bodies, resulting in less liver damage and neurological symptoms at the same level. However, it is uncertain if green tea protects against WD via complexing copper and its unique regulatory mechanism.

Green tea polyphenol (GTP) is a group of phenolic compounds and their derivatives found in green tea that have antioxidant and metal-chelating properties. It is believed that the aromatic hydroxyl groups in the compound, their relative positions, and the oxidation state of Cring enable them to complex copper ions [17]. Researchers have demonstrated that GTP can form stable complexes with copper, thereby reducing the concentration of free copper [18]. This complexation not only decreases copper toxicity, but it may also protect cells from oxidative damage by reducing copper-induced oxidative stress [19]. In addition, the green tea-specific amino acid L-theanine (L-TA) has been reported to have protective effects on alcoholic liver injury, lipopolysaccharide induced inflammation, and acute liver injury [20,21,22]. By chelating copper, L-TA was able to directly block EGCG oxidation and hydroxyl radical generation, significantly reducing hepatotoxicity [23]. In vitro data demonstrated that L-TA and copper generated an L-TA–copper complex, which inhibited copper’s redox activity [24]. A better understanding of these pathways may help to investigate the health benefits of GTE and its possible use in the treatment of metal-induced oxidative stress.

In this study, we examined for the first time the copper excretion and hepatoprotective effects of GTE in TX mice, focusing on tissue copper accumulation and hepatic oxidative stress injury. We utilized the levels of L-TA and polyphenolic components as indicators and identified compounds with superior complexing ability with Cu^2+^ through quantitative analysis of L-TA and polyphenolic content, as well as assessment of their complexation capacity with Cu^2+^ in GTE. The copper detoxification and hepatoprotective actions of GTP and L-TA were ultimately confirmed at the animal level.

## 2. Materials and Methods

### 2.1. Chemicals and Reagents

Aminotransferase (AST), alanine aminotransferase (ALT), alkaline phosphatase (AKP), superoxide dismutase (SOD), malondialdehyde (MDA), glutathione (GSH), and a Bradford Protein Concentration Measurement Kit were obtained from the Institute of Biological Engineering (Nanjing, China). The hematoxylin and eosin dyes were obtained from Jining Biological Co., Ltd. (Shanghai, China). The theanine standard, gallic acid, catechin, epicatechin, gallocatechin, epigallocatechin, epicatechin gallate, epigallocatechin gallate, tea polyphenols, and L-theanine were purchased from Yuanye Bio-technology Co., Ltd. (Shanghai, China). Anhydrous ethanol, hydrogen peroxide, nitric acid, formic acid that was chromatographically pure, and sodium nitrate were obtained from Runjie Chemical Reagent Co., Ltd. (Shanghai, China). Paraformaldehyde (4%), ammonium tetrathiomolybdate, and penicillamine were obtained from Macklin Biological Co., Ltd. (Shanghai, China). Sodium pentobarbital and cupric sulfate were obtained from Kangwei Pharmaceutical Co., Ltd. (Henan, China). Sodium chloride solution (0.9%) was obtained from Sinopharm Co., Ltd. (Shanghai, China). Hepes buffer (1 mM) was purchased from Solepipe Technology Co., Ltd. (Beijing, China). Green tea extract was obtained from Ruicao Co., Ltd. (Shanxi, China). Acetonitrile was obtained from Aladdin Biochemical Technology Co., Ltd. (Shanghai, China).

### 2.2. Preparation of Green Tea Extract

We screened and crushed the initial green tea materials in a versatile stainless steel extraction tank. We then added an eight-fold amount of ultrapure water, followed by three cycles of reflux extraction. We filtered and combined the resulting liquid before concentrating it at a temperature of 70 °C. Lastly, the concentrate was freeze-dried to obtain green tea extract.

### 2.3. Ultra Performance Liquid Chromatography (UPLC) Conditions

Chromatographic separation was achieved with a Waters Acquity UPLC system (Waters, Milford, CT, USA). L-TA Chromatographic conditions: Analysis was achieved on the ZORBAX RRHD Eclipse Plus C18 column (2.1 × 100 mm × 1.8 µm, Agilent, Santa Clara, CA, USA), along with the detective wavelength of 199 nm. Mobile phase A: acetonitrile; Mobile phase B: 0.1% formic acid in water. The gradient program was as follows: 0–5 min, 2–2% A; 5–10 min, 2–80% A; 10–18 min, 80–2% A; 18–20 min, 2–2% A; at the flow rate of 0.25 mL·min^−1^, and the column temperature was maintained at 30 °C. The injection volume of the sample was 2 μL.

GTE Chromatographic conditions: Analysis was achieved on the ZORBAX RRHD Eclipse Plus C18 column (2.1 × 100 mm × 1.8 µm), along with the detective wavelength of 278 nm. Mobile phase A: acetonitrile; Mobile phase B: 0.1% formic acid in water. The gradient program was as follows: 0–30 min, 5–20% A; 30–32 min, 20–20% A; 32–33.5 min, 20–95% A; 33.5–35 min, 95–5% A; 35–42 min, 5–5% A; at the flow rate of 0.25 mL·min^−1^, and the column temperature was maintained at 30°C. The injection volume of the sample was 2 μL.

### 2.4. Experimental Animals Grouping and Handling

Mice with the toxic milk (TX) mutation have an 82% homologous gene to the human *ATP7B* gene and have a phenotype very similar to that of WD patients [25,26].

Dong Ting, Director of the Department of Encephalopathy at the First Affiliated Hospital of Anhui University of Traditional Chinese Medicine, graciously donated male and female four-month-old TX mice, as well as homozygous DL mice. The average body weight was 25 ± 5 g. Anhui Agricultural University’s SPF-grade animal laboratory raised these mice for passage, adhering to the 3R guidelines for laboratory animals. Furthermore, the current study gained ethical approval from Anhui Agricultural University’s Animal Ethics Committee Ethical (AUAU2021012).

**Figure 1:**
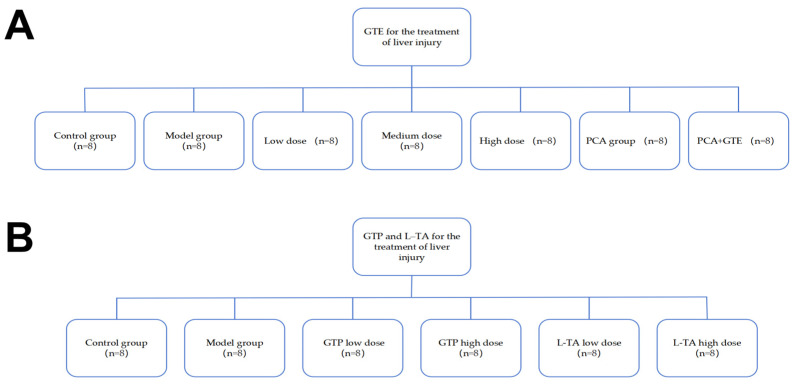
Experimental animal groupings. (**A**) Grouping of animals for experiments on GTE for treatment of liver injury; (**B**) Grouping of animals for experiments on GTP and L-TA for treatment of liver injury.

GTE for the treatment of liver injury was tested using 8 DL mice (blank control group) and 48 TX mice (Figure 1A). We randomly assigned the 48 TX mice to six groups: the model, GTE low-dose (600 mg/kg/day), medium-dose (800 mg/kg/day), high-dose (1000 mg/kg/day), PCA (100 mg/kg/day), and PCA + GTE (100 mg/kg/day + 800 mg/kg/day). Each group had eight mice, with an equal number of male and female individuals. The mice received 0.1 mL per g body weight intragastrically once a day for 30 days, while the blank and model control groups received similar quantities of saline via gavage.

GTP and L-TA for the treatment of liver injury was tested using 8 DL mice (blank control group) and 40 TX mice (Figure 1B). We randomly assigned the 40 TX mice to five groups: the model group, the GTP low-dose group (150 mg/kg/day), the GTP high-dose group (300 mg/kg/day), the L-TA low-dose group (150 mg/kg/day), and the L-TA high-dose group (300 mg/kg/day). Each experimental group consisted of eight mice, with an additional eight DL mice serving as blank control groups. For 30 days, we administered 0.1 mL per g of body weight intragastrically to the mice, while we gavaged 0.9% aqueous sodium chloride solution to the blank control and model groups.

### 2.5. Sample Collection

After being administered the final dose, groups of mice were placed in metabolic cages and exposed to fasting conditions without water restriction. Urine and feces were collected during the following 12 h period. We drew blood from the eye canthus of each group after anaesthetizing them with 1% sodium pentobarbital, while liver, kidney, and brain tissues were harvested as well. The blood and urine samples underwent centrifugation at a speed of 4000 r/min for 15 min before being stored at −80 °C in a refrigerator.

### 2.6. Preparation of Solutions

GTE and GTP sample solutions were prepared by weighing 50 mg of GTE and GTP powders in a 50 mL volumetric flask, followed by the addition of ultrapure water to achieve a concentration of 1.0 mg/mL for both GTE and GTP master batches.

The control solution included the following components: theanine (0.87 mg), gallic acid (0.85 mg), catechin (1.45 mg), epicatechin (1.45 mg), gallocatechin (1.53 mg), epigallocatechin (1.53 mg), epicatechin gallate (2.21 mg), epigallocatechin gallate (2.29 mg), and ammonium tetrathiomolybdate (1.30 mg). The ingredients were precisely measured, dissolved in ultrapure water, and adjusted to a final volume of 2 mL. Penicillamine was subsequently incorporated into the solution at a concentration of 37.25 mg/100 mL by adjusting the volume accordingly. A control master mix with a concentration of 2.5 mM was then prepared following the aforementioned steps and stored at 4 °C.

To prepare a 1 M NaNO_3_ solution, we weighed 4.25 g of NaNO_3_ and dissolved it in ultrapure water until it reached a final volume of 50 mL.

We weighed 0.8 g of CuSO_4_ and dissolved it in a 50 mL volumetric flask to prepare a standard solution of Cu^2+^. This solution was then diluted to create a series of concentrations: 10^−2^, 10^−3^, 10^−4^, 10^−5^, and 10^−6^ M. The electrode potential E (mV) of the copper standard solution was measured using an ion meter, and a standard curve was constructed by plotting E against −lgC_Cu_^2+^. The standard curve was constructed with E plotted against −lgC_Cu_^2+^.

### 2.7. Histopathology of the Liver

The liver tissues were collected from the same anatomical region in each group of mice. Then, randomly selected livers were fixed in 4% paraformaldehyde for more than 24 h, dehydrated with ethanol, embedded in paraffin, sectioned, and stained with hematoxylin and eosin. Finally, the liver sections of the mice were examined under an optical microscope.

### 2.8. Determination of Serum and Liver Biochemical Parameters

Serum samples were prepared and used to measure the ALT, AST, and AKP levels per the instructions provided in their respective assay kits. A 20% liver homogenate was also prepared by adding pre-cooled saline at a mass volume ratio of 1:4 (mg:μL). SOD, MDA, and GSH levels were determined using the protocols outlined in the corresponding kit instructions. The Bradford protein concentration assay kit measured the total liver protein content. Outcome assessments were performed in a blinded manner.

### 2.9. Determination of Copper Content in Liver, Brain, Kidney, Urine, and Feces

We collected a 0.2 g liver tissue sample, rinsed it with physiological saline, and gently dried it by blotting. We then added a 4 mL solution of nitric acid and allowed it to react for 30 min. Next, we added 1 mL of H_2_O_2_ and heated the mixture on a hot plate until the liver tissue was completely digested. Once the digestion was complete, we reheated the acid solution to encourage evaporation and then cooled it to room temperature. We quantified the hepatic copper content using atomic absorption spectrophotometry after adjusting the volume to 25 mL with a 1% HNO_3_ solution. We processed the mice’s brains, kidneys, and feces using the same procedure as that for the liver. We adjusted the fecal samples to a final volume of 10 mL using 1% HNO_3_. For urine analysis, we combined 1 mL with 24 mL of diluent (0.5% HNO_3_–0.1% Trillatone), vortexed it for 30 s, and then centrifuged it at low temperature (4000 r/min) for 15 min before measurement.

### 2.10. Analysis of the Complexing Ability of the Compounds with Cu^2+^

A quantity of 1.0 mL each of GTE, GTP, L-TA, and each polyphenol and chemical monomer were individually introduced to a 0.1 mM Cu^2+^ standard solution, maintaining an ionic strength of 0.01 M and utilizing a 10 mM Hepes buffer solution at pH 7.2 to 7.4. The solution was agitated at a consistent temperature of 37 °C. After reaching equilibrium, we introduced a copper-ion selective electrode and a reference electrode into the solution, documenting the measurements before and after the reaction. The E values of several compounds were documented, and the rate of Cu^2+^ complexation was determined using the following formula.

CR (%) = (A_0_ − A_1_)/A_0_ × 100% (1)

in which CR is the Cu^2+^ complexation rate, A_0_ is the Cu^2+^ initial concentration, A_1_ is the Cu^2+^ concentration after complexation.

### 2.11. Statistical Analysis

All experiments were repeated three times, and the results are expressed as mean ± standard deviation. Statistical analysis was performed using GraphPad Prism 8.0.2 software. Two independent sample *t*-tests were used to compare two groups, and a one-way analysis of variance (ANOVA) was used to compare multiple groups.

## 3. Results

### 3.1. Green Tea Extract Attenuated the Pathological Changes in the Liver of TX Mice

**Figure 2:**
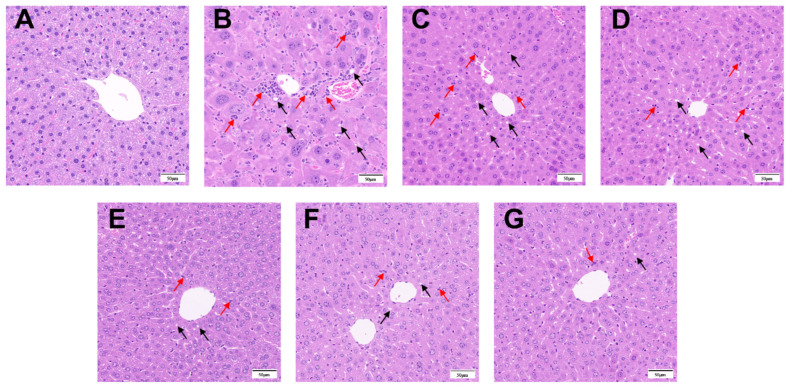
HE staining of liver pathological sections of mice in different groups (×200). (**A**) Control group; (**B**) Model group; (**C**) Low-dose GTE group; (**D**) Medium-dose GTE group; (**E**) High-dose GTE group; (**F**) PCA group; (**G**) GTE + PCA group (Black arrow: cellular vacuolation; Red arrow: glycogen nucleus).

After administration of GTE intervention to TX mice, changes in the morphology and structure of hepatocytes were observed by hematoxylin-eosin staining (HE). The histopathological observations in Figure 2A–G revealed that the control group exhibited a standard hepatocyte structure characterized by a distinct outline and orderly arrangement (Figure 2A). In contrast, the model group displayed a significantly deteriorated hepatocyte structure with a disorganized arrangement, increased cell vacuolation (indicated by black arrows), cell necrosis (indicated by red arrows), and an augmented presence of aggregated ribosomes (Figure 2B). Increases in the administered dose of GTE significantly improved the liver’s abnormal structural disorganization and reduced cellular debris (Figure 2C–E). The positive control drug PCA reduced hepatocyte vacuolation and necrosis in TX mice, although cell boundaries remained indistinct (Figure 2F). Hepatocyte injury was substantially diminished in the GTE + PCA group, with minimal histological abnormalities resembling those of the standard group (Figure 2G).

### 3.2. Green Tea Extract Improved Liver Function and Reduced Oxidative Stress in TX Mice

**Figure 3:**
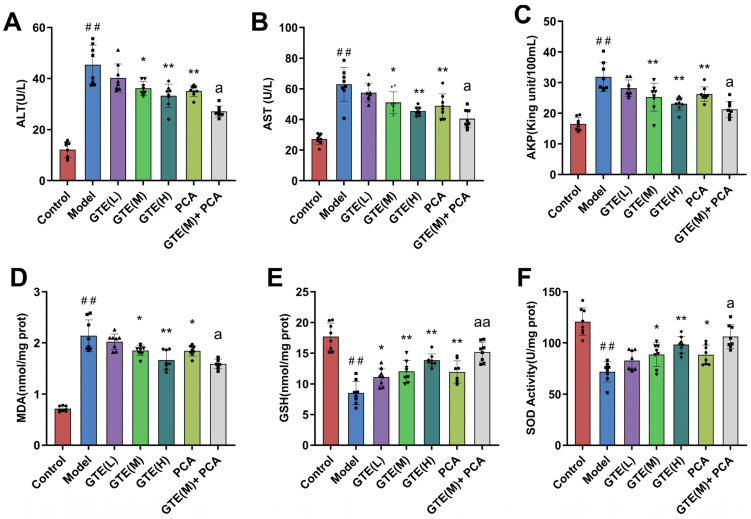
Effects of GTE and PCA on AKP (**A**), ALT (**B**), AST (**C**) activities in the serum and MDA (**D**), GSH (**E**) levels and SOD activity (**F**) in the liver ($\bar{x}\pm S$, *n* = 8). Statistical significance: # *p* < 0.05 and ## *p* < 0.01 compared with the control group, and * *p* < 0.05 and ** *p* < 0.01 compared with the model group. *a p* < 0.05 and *aa p* < 0.01 compared with the PCA group. (The number of these dots, frames, and triangles represents the number of samples in each group, and they are shaped to differentiate between the different groups, same below).

By analyzing serum levels of AST, ALT, AKP, and other markers, we investigated the hepatoprotective effects of GTE and its characteristic components in mice induced by copper overdose. As depicted in Figure 3A–C, compared to the standard control group, the model group exhibited significantly elevated serum ALT, AST, and AKP activities (*p* < 0.01). Different doses of GTE reduced the activities of all three enzymes to varying degrees (*p* < 0.05, *p* < 0.01). The combined administration of GTE and PCA showed superior efficacy in treatment (*p* < 0.05). Liver indices were subsequently examined to investigate whether green tea has an anti-oxidative stress effect. As shown in Figure 3D–F results, the MDA level of mice in the model group was significantly higher than that of the control group (*p* < 0.01), and the levels of SOD and GSH were substantially lower than those of the control group (*p* < 0.01). Different doses of GTE and PCA exhibited varying degrees of improvement in hepatic enzyme metabolism and inhibition of lipid peroxidation (*p* < 0.05, *p* < 0.01), with better effects observed for the combination of GTE and PCA (95% CI 0.003798–0.5068, −5.890–−0.6093, −34.22–−1.461, *p* < 0.05, *p* < 0.01, *p* < 0.05).

### 3.3. Green Tea Extract Improved Copper Metabolism in TX Mice

**Figure 4:**
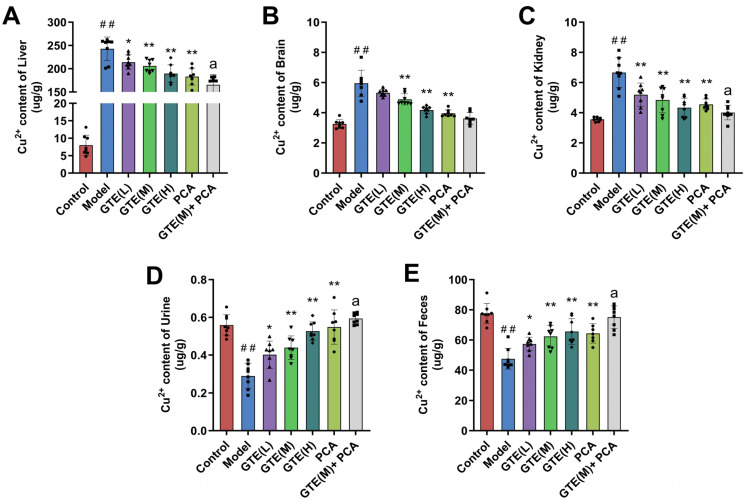
The effects of GTE and PCA on the copper level in the liver (**A**), brain (**B**), kidney (**C**), urine (**D**), and feces (**E**) of TX mice ($\bar{x}\pm S$, *n* = 8). Statistical significance: # *p* < 0.05 and ## *p* < 0.01 compared with the control group, and * *p* < 0.05 and ** *p* < 0.01 compared with the model group. *a p* < 0.05 and *aa p* < 0.01 compared with the PCA group.

In this experiment, copper levels in each mouse group’s liver, brain, kidney, urine, and feces were measured using the AAS method. As depicted in Figure 4A–C, the model group exhibited significantly elevated liver, brain, and kidney copper levels (*p* < 0.01). Consistent with clinical observations, hepatic copper accumulation was particularly pronounced. Conversely, urinary and fecal copper levels were markedly lower in the model group compared to the standard group (Figure 4D,E, *p* < 0.01), suggesting impaired biliary excretion of copper due to a mutation at the *ATP7B* gene locus. Following treatment with varying doses of GTE and PCA compounds, there was a significant reduction in tissue copper levels within the liver, kidney, and brain of mice from the model group (*p* < 0.05, *p* < 0.01). Notably, combination therapy involving GTE and PCA demonstrated superior efficacy in promoting tissue-specific copper metabolism among TX mice subjects.

### 3.4. The Content of GTP and L-TA in GTE Complexation and the Ability of Their Representative Fractions to Complex with Cu^2+^

**Figure 5:**
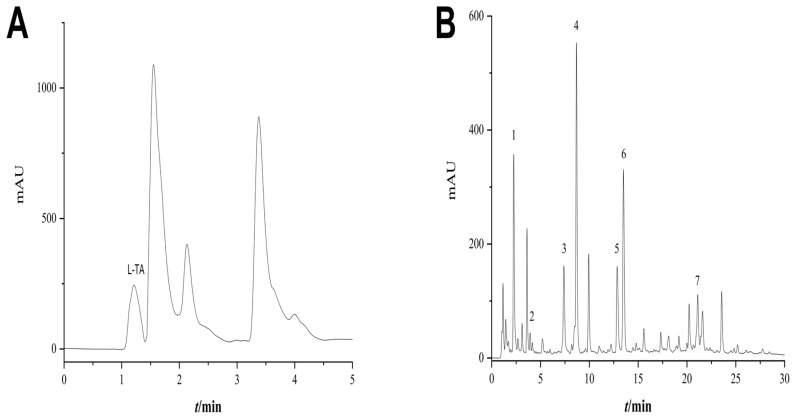
(**A**) UPLC chromatogram of L-theanine in green tea extract. (**B**) UPLC chromatograms of seven polyphenols in GTE, 1–7 are: GA, GC, EGC, C, EC, EGCG, ECG.

Based on the chromatographic peak areas shown in Figure 5A,B, we were able to determine the following content in the GTE sample: 16.624 mg/g L-TA, 6.202 mg/g GA, 12.445 mg/g GC, 50.470 mg/g EGC, 43.134 mg/g C, 13.767 mg/g EC, 20.636 mg/g EGCG, and 7.132 mg/g ECG. The green tea extract included 1.66% theanine and 15.38% tea polyphenol monomers such as GA, GC, EGC, C, EC, EGCG, and ECG.

According to Table 1, the complexing ability of each monomer compound is as follows: GA > EGCG > C > EC > ECG > EGC > CG. GTE, GTP, L-TA, polyphenol monomers, and chemical monomers all show high Cu^2+^ complexing abilities. This indicates that GTP and L-TA may be the key components of GTE, which play a crucial role in copper exclusion by complexing Cu^2+^.

### 3.5. GTP and L-TA Reduced Liver Pathology in TX Mice

**Figure 6:**
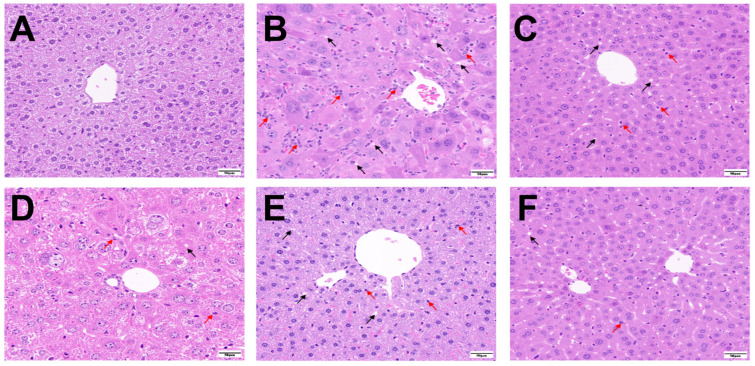
HE staining of liver pathological sections of mice in different groups (×200). (**A**) Control group; (**B**) Model group; (**C**) Low-dose GTP group; (**D**) High-dose GTP group; (**E**) Low-dose L-TA group; (**F**) High-dose L-TA group (Black arrow: cellular vacuolation; Red arrow: glycogen nucleus).

Histopathological examination of GTP and L-TA-treated livers from TX mice revealed no histological abnormalities in the control group (Figure 6A), whereas increased cytoplasmic vacuolization and cell necrosis were observed in the model group (Figure 6B). Hepatocellular injury was slightly improved in both the GTP and L-TA treatment groups when compared to the model group, confirming green tea’s protective effect on WD liver injury (Figure 6C–F).

### 3.6. GTP and L-TA Improved Liver Function and Reduced Oxidative Stress in TX Mice

**Figure 7:**
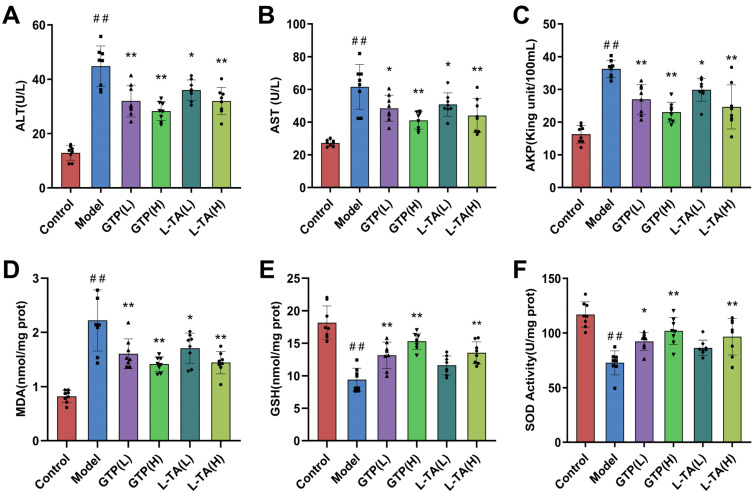
Effects of GTP and L-TA on AKP (**A**), ALT (**B**), AST (**C**) activities in the serum and MDA (**D**), GSH (**E**) levels and SOD activity (**F**) in the liver ($\bar{x}\pm S$, *n* = 8). Statistical significance: # *p* < 0.05 and ## *p* < 0.01 compared with the control group, and * *p* < 0.05 and ** *p* < 0.01 compared with the model group.

High-dose GTP therapy significantly reduced ALT, AST, and AKP serum levels in TX mice (Figure 7A–C; *p* < 0.01). Both low and high doses of L-TA significantly reduced serum ALT and AKP activity (*p* < 0.05; *p* < 0.01). Different doses of GTE and PCA improved hepatic enzyme metabolism and inhibited lipid peroxidation, with the combination of GTE and PCA having stronger benefits (*p* < 0.05, *p* < 0.01). Furthermore, as shown in Figure 7D–F, high doses of both GTP and L-TA increased SOD and GSH levels in mice while decreasing MDA levels (*p* < 0.01). These data indicate that GTE and its characteristic components reduce oxidative damage in the livers of TX mice. These findings imply that GTP and L-TA are important components of green tea that have antioxidant properties that prevent liver damage.

### 3.7. Green Tea Polyphenols and L-Theanine Improved Copper Metabolism in TX Mice

**Figure 8:**
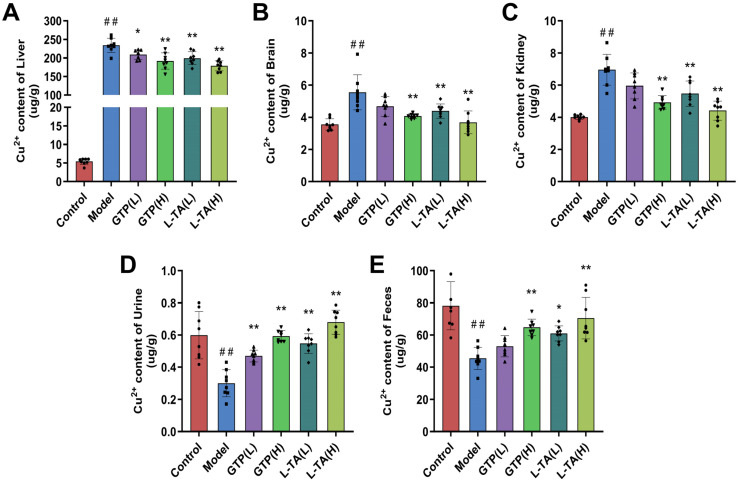
The effects of GTP and L-TA on the copper level in the liver (**A**), brain (**B**), kidney (**C**), urine (**D**), and feces (**E**) of TX mice ($\bar{x}\pm S$, *n* = 8). Statistical significance: # *p* < 0.05 and ## *p* < 0.01 compared with the control group, and * *p* < 0.05 and ** *p* < 0.01 compared with the model group.

Figure 8A–E shows that high doses of GTP and L-TA treatments dramatically lowered copper concentration in TX mice’s liver, kidney, and brain while boosting copper elimination through urine and feces (Figure 8, *p* < 0.01). At the total animal level, it was demonstrated that GTP and L-TA could complex excess copper in TX mice, enhance copper metabolism and excretion, minimize copper buildup in organs, and restore the organism’s copper homeostasis.

## 4. Discussion

Wilson’s disease is an autosomal recessive illness of copper metabolism caused by a mutation in the *ATP7B* gene at 13q14.3, resulting in defective copper cyanoprotein production and biliary excretion [27,28]. The *ATP7B* gene is mostly expressed in the liver and is responsible for copper transport in hepatocytes; therefore, excess copper ions in WD patients are not eliminated in the liver via bile but instead accumulate in the liver, eventually leading to cirrhosis and even liver failure [29]. At the moment, drugs used to treat WD are mostly focused on increasing copper excretion, lowering tissue free copper concentration, and reducing damage caused by too much copper through oxidative stress. However, avoiding the severe side effects associated with these medications remains difficult. Notably, penicillamine and other chelating drugs, including tetrathiomolybdate, may cause a potentially irreversible neurological deterioration as a result of a rapid rise in free copper [30]. Tea has been found to complex with heavy metal ions, thereby reducing the concentration of free metal ions, as reported in existing studies [31,32]. The purpose of this study was to look into GTE’s therapeutic role and method for treating liver injury in TX mice. Furthermore, we screened compounds with improved complexing ability with Cu^2+^ by measuring the concentrations of L-TA and polyphenolic components in GTE and evaluating their complexing ability with Cu^2+^. Finally, the copper exclusion and hepatoprotective effects of GTP and L-TA were tested in animals. Thus, the results of this investigation provide useful insights into the possible therapeutic application of GTE in WD liver injury.

The HE staining results showed that green tea may improve the shape and organization of hepatocytes and protect the liver from oxidative stress caused by copper. Elevated serum ALT, AST, and AKP levels typically suggest the presence of hepatocellular injury [33]. Serum ALT, AST, and AKP levels significantly decreased in TX mice following GTE treatment. Oxidative stress is responsible for the production of several reactive oxygen species (ROS). Antioxidant enzymes (such as SOD) and non-enzymatic antioxidants (such as GSH) are thought to be important causes of oxidative stress. SOD reduces the production of free radicals and lipid peroxides while also accelerating their clearance, minimizing hepatocyte damage [34]. GSH is essential for protecting cells from oxidative damage and regulating redox equilibrium [35]. SOD activity and GSH levels indirectly indicate the body’s ability to scavenge oxygen-free radicals. As a result, maintaining optimal amounts of SOD and GSH is essential for preventing and reducing oxidative stress damage in the body. The results showed that TX mice had significantly higher levels of hepatic MDA and lower levels of SOD and GSH activity. However, GTE treatment brought these levels back to normal or nearly normal levels. Also, AAS results showed that GTE effectively decreased copper buildup in the liver, brain, and kidneys while increasing copper elimination through urine and feces. Giving GTE along with PCA had a bigger impact on copper elimination activity and liver protection than giving either GTE or PCA alone. The above findings are consistent with prior research, which found that GTE had a positive effect on liver health. For example, a meta-analysis found that green tea drinking improved liver enzymes in patients with non-alcoholic fatty liver disease but had little effect in healthy people [36]. Furthermore, animal studies have demonstrated that green tea supplementation is effective in preventing perfluorodecanoic acid-induced hepatic oxidative stress, degeneration, and inflammation by analyzing serum, liver biochemical parameters, histological changes, and other relevant indicators [37]. These data supported the therapeutic potential of GTE in liver-related diseases.

Based on the findings, it is hypothesized that copper exclusion and antioxidant actions are important in GTE’s ability to ameliorate WD liver injury. However, further research is necessary to identify the specific components. As a result, we did quantitative analyses of eight active parts of GTE: L-TA, GA, C, EC, GC, EGC, ECG, and EGCG. These all have pharmacological effects in common. The results showed that GTE contained 1.66% of L-TA, while seven polyphenol monomers such as GA, GC, EGC, C, EC, EGCG, and ECG, accounted for 15.38% of the total GTE. To determine the individual components of GTE that produced copper exclusion and antioxidant effects, an ionometric screening approach for Cu^2+^ complexing capacity was developed. The screening of copper-complexing active components in green tea extracts revealed that GTP and L-TA have a high complexing activity for Cu^2+^. The superior complexing capability of the chemomonitor TM compared to PCA aligns with the clinically documented copper detoxifying benefits of WD medicinal agents and substantiates the efficacy of the ionometric approach for evaluating Cu^2+^ complexing capacity.

Previous research has revealed that GTP and L-TA have strong copper-complexing abilities and can form complexes with Cu^2+^, which may be the primary active components of green tea in increasing copper excretion in TX mice. The precise effects of GTP and L-TA on copper excretion and their capacity to mitigate WD liver damage remain ambiguous. That is why we looked into the copper excretion and liver-protecting effects of both drugs. Furthermore, neither L-TA nor GTP are harmful [38]. GTP did not cause acute or chronic toxicity in SD rats at a dose of 800 mg/kg/day [39]. Lv et al. discovered that giving different amounts of GTP greatly decreased the damage that acetaminophen (APAP) did to the liver and increased survival from APAP overdose, hepatocyte necrosis, and ALT/AST/GSH levels [40]. Sadzuka et al. [41] treated acute alcoholic liver injury in mice with an intraperitoneal injection of L-TA and found that L-TA inhibited alcoholic liver injury by preventing cellular lipid peroxidation. As a result, we supplied GTP and L-TA at dosages of 150 and 300 mg/kg, respectively. The experimental findings revealed that GTP and L-TA, at dosages of 150 and 300 mg/kg, could suppress the rise in serum ALT, AST, and AKP activities in TX mice. Additionally, HE staining revealed a decrease in liver injury in the mice. When compared to the control group, GTP and L-TA significantly decreased the level of hepatic MDA and boosted the activities of SOD and GSH in TX mice. Additionally, the AAS results demonstrated that GTP and L-TA could prevent cellular lipid oxidation, thereby inhibiting alcohol consumption. The AAS results showed that GTP and L-TA could also bind to free copper in living organisms and help remove copper from the liver, brain, and kidneys of TX mice through urine and feces, which reduced copper buildup. Interestingly, at the same dose, the copper-repellent activity of L-TA was greater than that of GTP, which was consistent with the results of the in vitro research on Cu^2+^ complexing ability, possibly due to the presence of additional Cu^2+^ binding sites in the structure of L-TA. L-TA may exhibit higher bioavailability in vivo, allowing for better absorption and distribution in the body compared to other compounds. Secondly, in vivo environments involve complex interactions within biological systems. L-TA may interact synergistically with other endogenous compounds or therapeutic agents, enhancing its overall efficacy. These interactions can lead to increased potency or improved mechanisms of action that are not observable in in vitro studies. These findings imply that GTP and L-TA’s copper-complexing capacity and antioxidant activity are important components of green tea’s ability to regulate copper homeostasis and redox balance in the body, as well as to mitigate WD liver injury.

In this investigation, we found that administering green tea extract was helpful in reducing copper buildup in TX mice while also providing hepatoprotective benefits. However, additional research is necessary to ascertain its mechanism of action. For example, it is critical to get a thorough understanding of the interaction mechanism between green tea components and copper ions in vivo, as well as their impact on hepatic cell signaling pathways. In addition, the short treatment course and small sample size may affect the robustness and statistical power of our results, and we will involve a larger cohort in future studies to validate our findings. We should also conduct a more in-depth investigation of the differences between murine models and human WD patients to determine whether green tea is suitable for different groups. Researchers can conduct clinical trials to investigate the therapeutic effects of green tea on liver ailments and its potential preventive effects on other chronic illnesses such as cardiovascular disease and cancer. Furthermore, to assure its safety, green tea’s potential side effects and safety concerns must be carefully considered.

## 5. Conclusions

In conclusion, the current investigation confirmed that GTE might reduce liver injury in TX mice and assessed the concentrations of GTP, L-TA, and seven polyphenols in GTE. Furthermore, GTP and L-TA were identified as unique GTE components with substantial Cu^2+^ complexing ability in vitro, and their antioxidant activity and copper detoxifying impact were validated using pharmacodynamics. Thus, this study emphasizes the particular importance of GTE in treating WD, while also presenting a novel therapeutic pathway for treating WD by copper complexation. However, the sample size of this study was small, and further studies are needed to investigate the differences between WD patients and model mice. We will involve a larger cohort in future studies to validate our findings.

## Figures and Tables

**Table 1:** Determination of chelating rate of different compounds with Cu^2+^ ($\bar{x}\pm S$, *n* = 3).

Samples	Potentiometric Value (Mv)	Complexation Ratio (%)
PCA	−201.5 ± 1.32	60.36
TM	−227.2 ± 2.76	96.98
L-TA	−223.1 ± 1.66	95.44
GA	−219.4 ± 2.41	93.40
GC	−210.4 ± 3.10	83.75
EGC	−210.7 ± 1.56	84.23
C	−215.8 ± 1.78	90.54
EC	−214.3 ± 1.34	89.00
EGCG	−217.2 ± 2.46	91.77
ECG	−212.9 ± 1.40	87.35
GTE	−214.8 ± 2.31	89.54
GTP	−218.7 ± 1.82	92.92

## Data Availability

The original contributions presented in this study are included in the article. Further inquiries can be directed to the corresponding author.

## Abbreviations

The following abbreviations are used in this manuscript:

WD	Wilson’s disease
GTE	Green tea extract
GTP	Green tea polyphenol
L-TA	L-theanine
UPLC-DAD	Ultra performance liquid chromatography
ROS	Reactive oxygen species
PCA	D-penicillamine
TM	Tetrathiomolybdat
HE	Hematoxylin-eosin staining
AST	Aspartate aminotransferase
ALT	Alanine aminotransferase
AKP	Alkaline phosphatase
SOD	Superoxide dismutase
MDA	Malondialdehyde
GSH	Glutathione
TX	Toxic milk
